# Recalcitrant Paraneoplastic Pemphigus Associated With Prostate Adenocarcinoma: Spectacular Evolution Under Androgen Deprivation

**DOI:** 10.7759/cureus.43585

**Published:** 2023-08-16

**Authors:** Hajar Moata, Fouzia Hali, Safia Zafad, Hassan Jouhadi, Soumiya Chiheb

**Affiliations:** 1 Department of Dermatology and Venerology, Ibn Rochd University Hospital, Casablanca, MAR; 2 Department of Oncology and Radiotherapy, Mohammed VI Cancer Treatment Center, Ibn Rochd University Hospital, Casablanca, MAR

**Keywords:** conventional immunosuppressive drugs, hormonotherapy, prostatic adenocarcinoma, stomatitis, paraneoplastic pemphigus

## Abstract

Paraneoplastic pemphigus (PNP) is a rare, autoimmune, blistering condition defined by severe stomatitis, polymorphous cutaneous eruptions, and underlying neoplasms. PNP associated with solid cancer is extremely rare. An association with prostate adenocarcinoma remains exceptional. We describe a 69-year-old patient with recalcitrant PNP associated with prostate adenocarcinoma showing spectacular response immediately after associating hormonotherapy with conventional immunosuppressive drugs.

## Introduction

Paraneoplastic pemphigus (PNP) is a rare, life-threatening condition. It is associated with polymorphic mucocutaneous manifestations with underlying neoplasia [1]. The pathogenesis of PNP is not yet completely elucidated [2]. It is mainly associated with lymphoproliferative neoplasias such as chronic lymphocytic leukemia, non-Hodgkin’s lymphoma, Castleman disease, and thymoma [3]. PNP remains exceptionally underreported in prostate cancer. We document a case of recalcitrant PNP revealing a prostatic adenocarcinoma in a 69-year-old male, rapidly regressing after associating hormonotherapy with conventional immunosuppressive therapy.

## Case presentation

**Figure 1:**
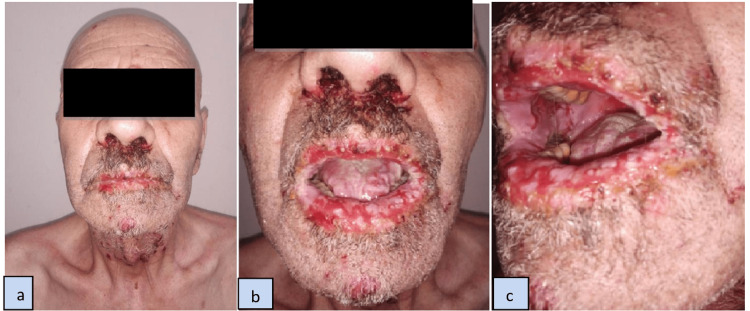
(a, b, c) Oral erosive mucositis, lips, and endonasal crusted erosions

A 69-year-old patient presented with severe oral erosive mucositis evolving for four months. He had no medical antecedents or family history of malignancy or autoimmunity. A physical examination also revealed crusting lesions of the lips, endonasal erosions (Figure 1), and marked conjunctivitis of the left eye with cutaneous erosions on the neck, upper trunk, and buttocks. Nikolsky’s sign, performed on oral mucosa and skin, was positive. A skin biopsy detected suprabasal epithelial detachment with a lymphocytic, eosinophilic, and neutrophilic infiltrate. Direct immunofluorescence showed positive fluorescence in the intercellular cement substance (ICS) of immunoglobulin G (IgG) while complement 3C, IgA, and IgM were negative. Indirect immunofluorescence showed an intercellular signal confined to the ICS, confirming the diagnosis of pemphigus vulgaris. The polymorphic mucocutaneous involvement, in particular of the oral mucosa, was suggestive of a PNP. Serum tumor markers, including prostate-specific antigen (PSA), alpha-fetoprotein, carcinoembryonic antigen (CA) 19-9, CA 125, and acid phosphatase were negative except for an elevated level of PSA (23.28 ng/ml; normal < 4.5). The patient underwent an abdominal and pelvic echography showing prostatic hypertrophy with calcifications.

To better assess the prostatic volume, a multiparametric prostatic MRI was performed that showed two prostatic lesions in the transitional zone that could be classified as Prostate Imaging Reporting and Data System (PI-RADS) 5, with a prostatic lesion in the peripheral zone classified as PIRADS 4. A prostatic biopsy revealed a conventional prostatic adenocarcinoma with a Gleason score of 8 (4+4). The patient was classified as a localized high risk, considering the digital rectal examination (classifying the patient as cT2c), the PSA value, and the Gleason score. Thoracic abdominal and pelvic computed tomography and bone imaging showed no significant abnormalities. Given the tumoral stage, a treatment based on hormonotherapy (luteinizing hormone-releasing hormone (LHRH) analogs) was recommended for 18 months.

**Figure 2:**
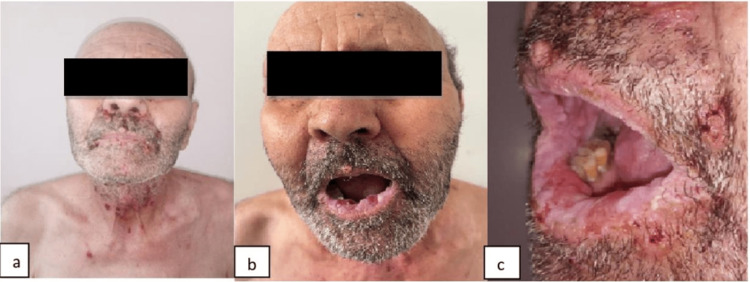
(a, b, c) Regression of erosive stomatitis and endonasal erosions after combining hormonotherapy with conventional immunosuppressive drugs

The patient was initially treated with prednisone 75 g/d and azathioprine 150 mg/d. After one month of immunosuppressive therapy, no improvement in dermatological symptomatology was noted. Hormonotherapy was then initiated. The patient was given subcutaneous injections of goserelin (10.8 mg/3 months). A spectacular regression of mucocutaneous erosions was observed (Figure 2) only one week after the first injection of hormonotherapy. Radiotherapy on the prostate, seminal vesicles, and lymph nodes was also indicated in this patient. No recurrence was noted after a one-year follow-up.

## Discussion

Prostate cancer is the second most common urological malignancy to be associated with paraneoplastic syndromes after renal cell carcinoma [4]. However, PNP associated with prostate cancer has rarely been described. To date, only two cases have been reported in the literature [5,6]. PNP, first described by Anhalt et al. in 1990, is the rarest and latest described subset of pemphigus [3].

The etiopathogenesis of PNP is not fully understood [7]. It seems that skin lesions are the result of an autoimmune response generated by antibodies directed against tumor antigens that cross-react with epithelial antigens mainly represented by periplakin and envoplakin. Cytokines (such as interleukin-6), which are produced and released by anti-tumor autoantibodies, stimulate the differentiation of B cells and promote the development of the humoral pathway of the immune system.

Stomatitis is the earliest and most constant clinical feature of PNP. It presents with erosions and ulcerations that can affect all surfaces of the oropharynx. Oral erosive mucositis is extremely resistant to therapy [8]. Cutaneous lesions are polymorphous. The blisters often erupt in waves, usually affecting the upper trunk, head, neck, and proximal extremities [9].

PNP is characterized by a lack of strictly specific histological criteria. A skin biopsy usually shows suprabasal acantholysis with scattered inflammatory infiltrates in the presence of blistering lesions while interface dermatitis and lichenoid dermatitis are more easily detected in the case of lichenoid or inflammatory skin lesions [10]. Direct immunofluorescence reveals IgG autoantibodies and/or complement deposition in the epidermal intercellular spaces and/or along the basement membrane zone. Indirect immunofluorescence on rat bladder epithelium is used to detect anti-plakin antibodies [1].

The treatment of PNP is based on the reduction of autoantibody production. Due to the poor prognosis, immunosuppressive therapy should be administered promptly. The first line of treatment is systemic corticosteroids with the addition of corticosteroid-sparing agents such as azathioprine, mycophenolate mofetil cyclosporine A. Rituximab, with or without concomitant intravenous immunoglobulin, is an interesting therapeutic option in case of failure of the first-line treatment [11]. It is important to treat neoplasia associated with PNP [3]. In our case, clinical improvement was observed only after combining hormonotherapy with conventional immunosuppressive therapy.

## Conclusions

PNP associated with prostatic adenocarcinoma is unusual. It is a challenging condition because its etiopathogenesis remains not fully elucidated, and conventional immunosuppressive drugs show poor effectiveness. Androgen deprivation may be considered an efficient adjuvant therapy of PNP in men presenting an underlying prostatic adenocarcinoma. However, further studies are needed to prove the efficiency of this use.
